# High frequency of lobular breast cancer in distant metastases to the orbit

**DOI:** 10.1002/cam4.331

**Published:** 2014-10-30

**Authors:** Mieke Raap, Wiebke Antonopoulos, Maximilian Dämmrich, Henriette Christgen, Diana Steinmann, Florian Länger, Ulrich Lehmann, Hans Kreipe, Matthias Christgen

**Affiliations:** 1 Institute of Pathology, Hannover Medical SchoolHannover, Germany; 2 Department of Radiation Oncology, Hannover Medical SchoolHannover, Germany

**Keywords:** Immunophenotype, lobular breast cancer, meta-analysis, metastasis, orbit

## Abstract

Metastasis to the periocular soft tissue of the orbit is a rare manifestation of metastatic cancer. Infiltrating lobular breast cancer (ILBC) is a special breast cancer subtype, which accounts for 10–15% of all mammary carcinomas and for ∼1% of all malignancies. Here, we report on a high frequency of lobular breast cancer in patients with orbital metastases identified in an original series of metastatic tumor specimens and by a systematic literature review. A series of 14 orbital metastases was compiled from formalin-fixed paraffin-embedded archival tissues. All cases were subjected to histological re-review and detailed immunophenotypical characterization. In addition, we performed a meta-analysis of 68 previously published case reports describing orbital metastases, with special reference to breast cancer subtypes. Based on clinical history, histomorphology, immunophenotype, and/or comparison with matched primary tumors, orbital metastases were derived from breast cancer in 8/14 cases, seven of which were classified as metastatic lobular breast cancer. Other entities included non-small cell lung cancer (4/14), infiltrating ductal breast cancer (1/14), prostate cancer (1/14) and adenocarcinoma of the esophagus (1/14). In line with this original series of orbital metastases, lobular breast cancer was the most common malignancy in 72 patients with orbital metastases described in 68 independent case reports. In conclusion, lobular breast cancer represents the cancer subtype with the highest prevalence among orbital metastases. The high frequency of ILBC in orbital metastases illustrates the special metastatic behavior of this tumor entity and may have implications for the understanding of the organotropism of metastatic lobular breast cancer.

## Introduction

Metastasis to the periocular soft tissue of the orbit is a rare manifestation of systemic cancer 1. Symptoms include ptosis, eye lid swelling and diplopia. Often, the possibility of an inflammatory process is raised 1. Computed tomography or magnetic resonance imaging may show a thickening of the extraocular muscles or an ill-defined orbital mass 1. In the context of a clinical history of cancer, an excision biopsy often confirms the seemingly improbable diagnosis of an orbital metastasis (OM).

There are no solid statistical data concerning the frequency of OMs in cancer patients. Shields and colleagues have reported 100 cases encountered at the Jefferson University, Philadelphia, within a period of 25 years 1. Henderson et al. have reported 83 cases seen at the Mayo Clinics, Rochester, in 40 years 2. Valenzuela et al. have described 80 cases collected from 4 Australian centers, seen in 22 years 3. This implies that histologically confirmed OMs are diagnosed once or twice per year at larger clinical centers. Accordingly, with exception of the three studies mentioned above, the literature on OMs is mainly restricted to case reports describing single cases. Of note, statistics on OMs do not arise from autopsy series, since enucleation of the bulbus oculi is no routine procedure during necroscopy.

One of the main clinical interests in OMs has been to define, which types of tumors spread to the orbit. Breast cancer has been identified as the most common malignancy involving this site. Depending on the series, breast cancers account for 29–53% of OMs 1–3. Albeit the histological and molecular diversity of mammary carcinomas is well known, breast cancer subtypes have never been taken into consideration in these studies 1–3.

Infiltrating lobular breast cancer (ILBC), is a special breast cancer subtype. ILBCs account for 10–15% of all mammary carcinomas and for ∼1% of all malignancies 4. ILBCs display a distinct histomorphology and are almost always estrogen receptor (ER)-positive 5. Contrary to infiltrating ductal breast cancers (IDBCs), which account for the vast majority of all mammary carcinomas, ILBCs are associated with inactivation of the tumor suppressor gene E-cadherin 4. ILBCs are slowly progressive tumors driven by estrogenic growth stimulation 6. Nonetheless, ILBCs are capable of widespread metastatic dissemination. ILBCs are over-represented in primary metastatic mammary carcinomas 7,8. Even so, ILBC metastases can also arise very long (>20 years) after initial tumor diagnosis and up to one-third of ILBC patients experience such metachronous recurrences 9,10. Preferentially involved sites differ significantly from IDBCs and include the ovaries, gut, peritoneum, skin, meninges and bone 10–14.

In our diagnostic histopathology service at the Hannover Medical School, we have recently noted two similar cases of ILBC metastatic to the orbit, one of which had been included in our previous study on lobular breast cancer progression 15. The repeated observation of this specific clinicopathological constellation prompted us to review archival OMs, and to perform a systematic literature review with special reference to breast cancer subtypes in OMs.

## Materials and Methods

### Tumor specimens

Formalin-fixed paraffin-embedded (FFPE) tumor specimens were retrieved from the tissue archive of the Institute of Pathology of the Hannover Medical School (MHH) according to the guidelines of the local ethics committee. Diagnostic reports (2000–2013) were screened for the diagnosis of OMs. Haematological malignancies and cases suspicious for a primary neoplasia of the orbit were excluded. Direct histomorphological comparison with the corresponding matched primary tumor (PT) was performed for all cases with available PT tissue. All specimens were made anonymous for batch-based immunohistochemical analysis and further scientific purposes. All cases of OMs were re-reviewed on HE- and PAS-stained full sections and immunohistochemical stainings by four histopathologists, who were blinded to the diagnostic considerations of their colleagues. Three of the four histopathologists were also blinded to the background of the study and any information available in the archival diagnostic reports, except for the distinction of PT or non-PT specimens, and were asked to determine the tumor type, histological subtype, and grade.

A control group of PTs was randomly selected from archival diagnostic reports using the following criteria: (1) tissue obtained in the same observation interval (2000–2013), (2) surgical resection specimen (punch biopsies were excluded), (3) PT (local recurrences were excluded), (4) therapy-naïve status (cases with known neoadjuvant therapy were excluded), (5) internal case ID starting with the digits 10–15, to limit the number of findings for a convenient sample size of eventually *n* = 134 cases.

### Immunohistochemistry

Immunohistochemistry was performed on a Benchmark Ultra (Ventana, Tucson, AZ) automated stainer using the CC1 mild protocol for antigen retrieval and monoclonal antibodies against E-cadherin (clone ECH-6, Zytomed Systems, Berlin, Germany, 1:100), *β*-catenin (clone 14, Beckton Dickinson, Heidelberg, Germany, 1:100), CK7 (clone OV-TL12/30, DAKO, Glostrup, Denmark, 1:100), Epithelial membrane antibody (EMA, clone E29, DAKO, 1:600), ER (clone SP1, Ventana, undiluted ready-to-use), BCL2 (clone 124, DAKO, 1:100), GATA binding protein 3 (GATA3, clone L50-823, BioCare Medical, Concord, CA, 1:200), mammaglobin (MGBN, clone 304-1A5, Biologo, Kiel, Germany, 1:10) and v-erb-b2 avian erythroblastic leukemia viral oncogene homolog 2 (ERBB2, clone 4B5, Ventana, undiluted ready-to-use). Detection of the immune reaction was achieved with the ultraView DAB kit (Ventana).

### Molecular genetic analysis

DNA was isolated from macro-dissected FFPE tumor specimens as described previously 15. Amplicon library preparation was performed using the Ion AmpliSeq Library kit 2.0, as recommended by the manufacturer (Life Technologies, Darmstadt, Germany; Carlsbad, CA). Briefly, a multiplex polymerase chain reaction (PCR) was done using 10 ng DNA and the Ion AmpliSeq colon and lung cancer panel v2. After ligation of barcoded sequencing adapters (Ion Xpress; Life Technologies), the final library was purified using AMPure beads (Beckman Coulter, Krefeld, Germany; Brea, CA) and was quantified by qPCR (Ion Library Quantification kit; Life Technologies). After dilution to a final concentration of 100 pmol/L, libraries were pooled and processed to library amplification on Ion sphere particles (ISPs) using Ion OneTouch chemistry (Life Technologies). Sequencing was performed using the Ion PGM 200 Sequencing kit v2 (Life Technologies) and 314 v2 chips (Life Technologies). Sequencing data from the PGM runs were processed with Ion Torrent Suite software version v4.0.2 (Life Technologies) and the variant caller v4.0 (Life Technologies).

### Meta-analysis

For meta-analysis of published cases reports, PubMed entries (2008–2013) were screened for the keyword “orbital metastasis”. Relevant publications were listed according to histological tumor entity, as given in the full-text articles. Tables S2 and S3 provide an overview of the case reports included in the meta-analyses.

### Statistics

Statistical significance of the dissimilar proportions of ILBCs and non-ILBCs in OMs versus PTs and BCs and non-BCs in the original series of OMs versus OMs from the literature review was assessed using GraphPad Prism software (GraphPad Prism Software Inc., La Jolla, CA) and the chi square test.

## Results

### Patient characteristics

**Figure 1:**
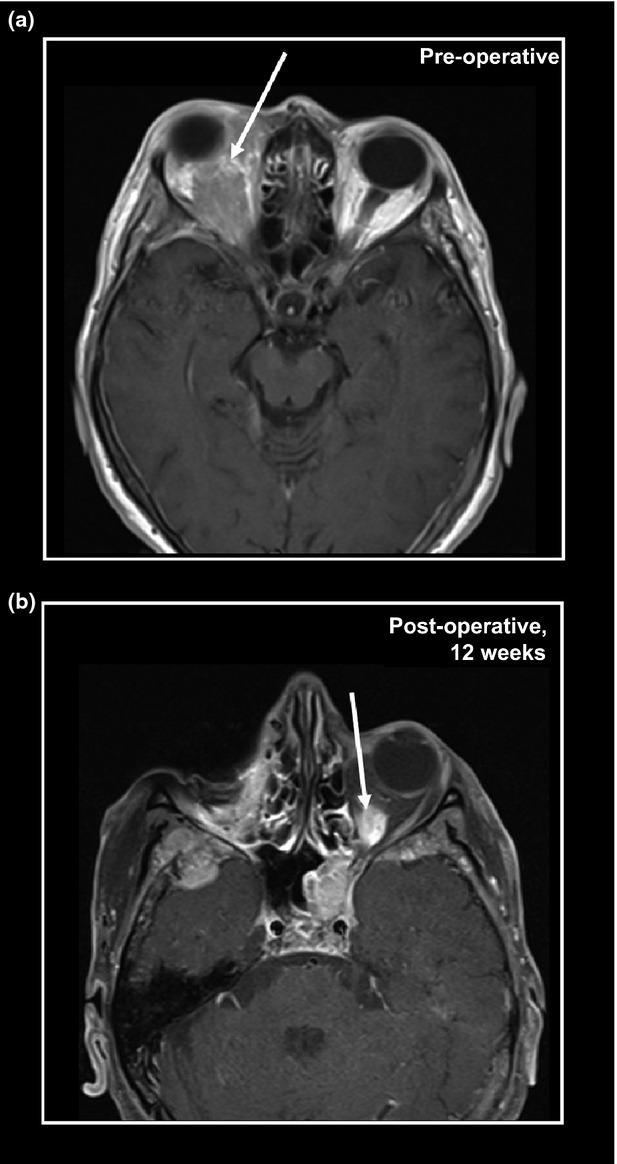
Magnetic resonance tomography (MRT) scans showing bilateral orbital metastases from infiltrating lobular breast cancer (ILBC). (A) MRT scan of a 61-year-old female patient, 11 years after bilateral mastectomy for ILBC and adjuvant therapy with tamoxifen and chemotherapy (cyclophosphamide, methotrexate, fluorouracil). The arrow indicates an ill-defined orbital tumor and exophthalmos. The patient underwent enucleation of the right bulbus oculi and resection of the periocular soft tissue, which confirmed a metachronous metastasis from ILBC. Subsequently, the patient was diagnosed with newly emerging hepatic metastases and was treated with second-line chemotherapy (paclitaxel). Twelve weeks after the enucleation of the right bulbus oculi, the patient was diagnosed with a newly emerging metastasis in the contralateral orbit. (B) MRT scan of the same patient, 12 weeks after enucleation of the right orbit. The arrow indicates an ill-defined lesion in the left orbit, suspicious for metastatic involvement.

In a period of 14 years, 14 orbital metastases (OMs) were diagnosed by histological evaluation at our institution (Table1). Median patient age was 67 years. Most patients (8/14) had a clinical history of breast cancer. Other entities included non-small cell lung cancer (4/14), adenocarcinoma of the esophagus (1/14) and prostate cancer (1/14). Histological subtypes of PTs were known for 9/14 cases. PTs included four ILBCs, two of which had been seen in our institution (Fig. S1). Four other primary carcinomas of the breast were of unknown histological subtype (Table1). The time to metastatic progression varied widely. In 5/14 patients PTs and OMs were diagnosed synchronously. All other OMs emerged metachronously. One OM from ILBC appeared 11 years after PT diagnosis. Two patients with metastatic breast cancer showed bilateral orbital involvement (Table1 and Fig.1).

**Table 1:** Patient characteristics

		Primary tumor				Metastasis		
	Age	Site	Histology	FFPE	Interval	Site	Histology^1^	FFPE
Patient 1	80	Breast	ILBC	–	Meta, na	Orbit	AC (ILBC), G2	av
Patient 2	51	Breast	Unknown	–	Meta, na	Orbit	AC (ILBC), G2	av
Patient 3	58	Breast	ILBC, G2	av	Syn	Orbit	AC (ILBC), G2	av
Patient 4^2^	61	Breast	ILBC, G2	av	Meta, 11 years	Orbit	AC (ILBC), G3	av
Patient 5	61	Breast	Unknown	–	Meta, na	Orbit	AC (ILBC), G3	av
Patient 6	80	Breast	ILBC	–	Meta, na	Orbit	AC (ILBC), G2	av
Patient 7^2^	63	Breast	Unknown	–	Meta, na	Orbit	AC (ILBC), G2	av
Patient 8	73	Breast	Unknown	–	Meta, na	Orbit	AC (IDBC^3^), G3	av
Patient 9	52	Esophagus	AC	av	Syn	Orbit	AC (NOS), G3	av
Patient 10	71	Lung	NSCLC, AC (NOS), G3	av	Syn	Orbit	AC (NOS), G3	av
Patient 11	67	Lung	NSCLC, ASQC, G3	av	syn	Orbit	ASQC, G3	av
Patient 12	76	Lung	NSCLC, AC (MT), G2	av	Syn	Orbit	AC (NOS), G2	av
Patient 13	68	Lung	NSCLC, AC (MT), G2	av	Meta, na	Orbit	AC (NOS), G2	av
Patient 14	70	Prostate	Unknown	–	Meta, na	Orbit	UC	av

AC, adenocarcinoma; ASQC, adenosquamous carcinoma; FFPE, formalin-fixed paraffin-embedded; av, FFPE available for histological analysis; ILBC, infiltrating lobular breast cancer; IDBC, infiltrating ductal breast cancer; Meta, metachronous; MT, mixed type; na, no specific information regarding the year of the primary tumor; NOS, not otherwise specified; NSCLC, non-small cell lung cancer; Syn, synchronous; UC, undifferentiated carcinoma.

1 The tumor subtype suggested by the histological appearance of the metastasis is indicated in parenthesis.

2 Biltateral orbital involvement.

3 The histological subtype of the OM of patient 8 was controversial among the reviewing pathologists, for details see text and Table S2.

### Histological and molecular findings in OMs

**Figure 2:**
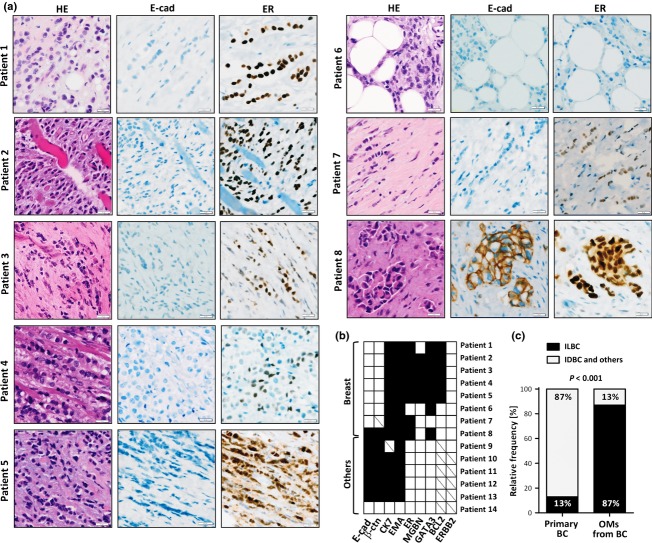
(A) Representative photomicrographs of orbital metastases (OMs) from patients with a history of breast cancer. HE-stained sections are shown on the left side (400×), immunohistochemical stainings for E-cadherin (E-cad) and estrogen receptor (ER) are shown on the right side. (B) Overview showing the immunophenotypical characteristics of the 14 OM. Immunohistochemical markers are aligned along the bottom, primary tumor sites along the left side. Filled squares indicate positive staining. Empty squares indicate negative staining. Diagonal lines indicate missing data. (C) Proportion of infiltrating lobular breast cancer (ILBC) in a control group of randomly selected primary breast cancers (BC, *n* = 17/134) diagnosed at our institution, compared with the proportion of ILBC in OMs from patients with a history of breast cancer (*n* = 7/8). Statistical significance was assessed with the chi-square test using the actual numbers of observed cases.

Histologically, OMs were adenocarcinomas, except for one undifferentiated carcinoma (PT of the prostate) and one adenosquamous carcinoma (PT of the lung) (Table1). Interestingly, 7/8 OMs from patients with breast cancer were histologically consistent with metastatic ILBC (Fig.2A, patient 1–7). These cases showed discohesive tumor cells, which were diffusely scattered or loosely packed or formed single-file linear cords, while infiltrating the periocular soft tissue. Four histopathologists independently classified these seven OMs as metastatic ILBC, and thus confirmed the original diagnoses documented in the archival reports (Table S1). Morphological comparison with the matched PT of patient 4, a grade 2 ILBC of the classic type, showed that the metachronous metastatic specimens had acquired a higher histological grade. The metastatic tumor cells exhibited a marked nuclear atypia, a tendency toward solid growth in loosely packed sheets of cells and a higher mitotic activity. This is consistent with transition to a secondary pleomorphic phenotype, as described previously for ILBC metastases 16. In line with their histomorphological features, these seven OMs were E-cadherin-negative and *β*-catenin-negative, while being positive for CK7 and EMA (Fig.2B, patient 1–7). Moreover, these OMs were commonly positive for ER or other mammary epithelial markers associated with luminal differentiation, such as MGBN, GATA3 and BCL2, and were ERBB2-negative, which is characteristic for ILBC (Fig.2B, patient 1–7) 17. In patient 3, concordant activating mutations in the *PIK3CA* oncogene (H1047L) in both, the PT and the OM, further supported that the OM was derived from lobular breast cancer.

The classification of the OM from patient 8, a female with a clinical history of breast cancer of unknown subtype, was controversial (Table1). This OM displayed small but pleomorphic tumor cells with eosinophilic cytoplasm and occasional signet ring cells. The growth pattern ranged from well-circumscribed, angulated patches to single-file linear cords and diffuse infiltration. Metastatic signet ring carcinoma of the stomach was raised as a differential diagnosis, but immunohistochemistry demonstrated expression of GATA3 and ER, which essentially excludes a gastric adenocarcinoma 18. Despite the partially discohesive growth pattern, tumor cells were strongly positive for E-cadherin, as usually seen in IDBC and metastases from IDBC (Fig.2A, patient 8) 19. This case was classified as metastatic IDBC or breast cancer of no special type by 3/4 involved breast histopathologists. The fourth observer favored a metastatic pleomorphic ILBC (Table S1). Eventually, a consensus was reached to classify this case as a metastatic IDBC (Table1).

### Clinical management

OM has a dismal prognosis and clinical management may include local irradiation, surgical resection, endocrine therapy or chemotherapy 3. No systematic clinical follow-up data were available for the patients corresponding to the tumor specimens of this retrospective histopathological study. Based on what could be reconstructed from clinical information provided in the histopathological data files, three of eight patients (3/8) with OMs and a history of breast cancer were treated with irradiation of the orbit (total dose 36–40 Gy) and endocrine therapy. Two patients received endocrine therapy only (tamoxifen or fulvestrant). Another patient with primary metastatic ILBC received chemotherapy (epirubicine/cyclophosphamide) and endocrine therapy. For two patients, further clinical management remained unknown. Radical surgery was attempted in one patient, who underwent enucleation of the right bulbus oculi and resection of the periorbital soft tissue (Fig.1). Subsequently, this patient developed a contralateral OM and metastases in the skull, cerebrum, cerebellum, and sinus cavernosus. Further treatment of this patient included irradiation and endocrine therapy, as stated above.

### Statistical considerations

Based on clinical history, histomorphology, immunophenotype, and/or comparison with the matched PTs, OMs were derived from breast cancer in 8/14 cases, seven of which were metastatic ILBCs. Next, we compared the proportion of ILBC in OMs from patients with a history of breast cancer with the proportion of ILBC in PTs of the breast diagnosed at our institution. Hence, we randomly selected a control group (surgical resection specimens diagnosed with breast cancer), from the same observation interval (see Materials and Methods for details). The frequency of ILBCs in this control group was consistent with the known prevalence of ILBC 4. As expected, comparison with our original series of OMs confirmed a statistically significant over-representation of ILBC in OMs compared with PTs (7/8, 87%, vs. 17/134, 13%, *P *< 0.001, chi square test) (Fig.2C).

### Meta-analysis of published case reports

**Figure 3:**
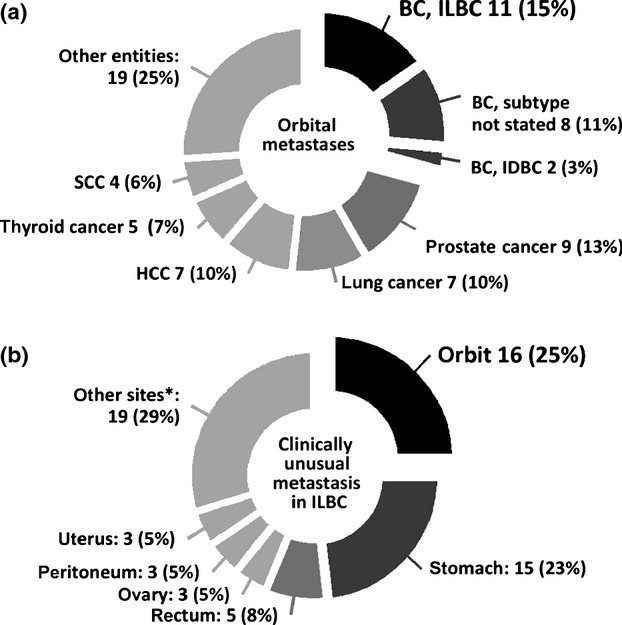
Meta-analysis of previously published case reports. (A) Orbital metastases (OMs) analyzed for primary tumor entities. BC, breast cancer; ILBC, infiltrating lobular breast cancer; IDBC, infiltrating ductal breast cancer; HCC, hepatocellular carcinoma; SCC, squamous cell carcinoma (head and neck, 1 SCC of the cervix uteri). Lung cancers included small cell and non-small cell lung carcinomas. Thyroid carcinomas included follicular thyroid carcinoma, papillary thyroid carcinoma, and medullary thyroid carcinoma. Other entities included: renal cell carcinomas (not further specified), colorectal cancer (adenocarcinomas and one signet cell carcinoma), melanoma, neuroendocrine tumors of the gastrointestinal tract, transitional cell carcinoma of the bladder, adenocarcinoma of the esophagus, chorioncarcinoma, gastric carcinoma (not further specified), gastrointestinal stroma tumor of the stomach, leiomyosarcoma of the abdominal cavity, Merkel cell carcinoma and an osteosarcoma of the tibia. (B) Anatomical sites of metastases from ILBCs, as described in previous publications. *Other sites included: Anus, aorta, bone, cervix uteri, kidney, meninges, liver, muscle, salivary glands, skin, and vulva.

To test whether our finding was representative, we performed a systematic meta-analysis of previously published case reports. This meta-analysis included 72 well-characterized OMs from 68 independent publications (Table S2, 2008–2013). The overall frequency of breast cancer was significantly lower in this data compilation compared with our original series (21/72, 29% vs. 8/14, 57%, *P *= 0.043, chi square test). Many case reports lacked information regarding the histological breast cancer subtype, as this was not always clinically relevant or reliably definable. Still, ILBC was the most commonly mentioned entity giving rise to OMs. In particular, OMs were attributed to ILBC approximately five times more often than to IDBC (Fig.3A).

Metastatic ILBC is notorious for a peculiar organotropism involving a variety of more or less unexpected anatomical sites 9,11–14. For completeness, we also reviewed publications describing clinically unusual cases of metastatic ILBC. This second meta-analysis included 64 distant metastases in 63 patients from 56 publications (Table S3, 1998–2013). The two most commonly reported sites were the stomach and the orbit, reflecting that these sites were probably least expected by the clinicians (Fig.3B).

## Discussion

Cancer metastasis to the orbit is rare. Few series of histological confirmed OM have ever been reported 1–3. Breast cancer has consistently been the most common malignancy in patients suffering from OMs 1–3. However, breast cancer is a histologically and biologically heterogeneous disease. Different breast cancer subtypes have different metastatic capabilities 11–14. For instance, brain-metastasizing breast cancers belong to the basal or ERBB2-positive subtypes 19. Despite this well-known heterogeneity, breast cancer subtypes have never been taken into consideration in studies on OMs 1–3.

This is the first study on breast cancer subtypes in OMs. All histologically confirmed OMs diagnosed at the Hannover Medical School within the last 14 years were subjected to a histological re-review and a detailed immunophenotypical characterization. OMs were derived from breast cancer in 8/14 cases, seven of which were classified as metastatic ILBC. In view of the relative rarity of primary ILBC (∼1% of all malignancies and 10–15% of all mammary carcinomas), this is a remarkable and statistically significant over-representation of ILBC in OMs. A systematic meta-analysis of previously published case reports appears to support this finding. ILBC was the most common malignancy in 72 patients with OMs described in 68 independent case reports.

This work has two overt clinical implications. First, orbital symptoms in patients with a known history of ILBC are particularly suspect for a metastasis, even if the primary diagnosis is more than a decade ago. Second, pathologist confronted with intraoperative biopsy evaluation of tumor processes in the orbit should bear in mind that metastatic ILBC is common at this site and can be misinterpreted in fresh frozen sections, due to sparse cellularity and discohesive growth.

Concerning tumorbiological characteristics, it is tempting to ask why ILBC is over-represented in OMs. The answer to this question remains open. However, keeping in mind that ILBC growth is strongly dependent on estrogenic stimulation 6, the list of anatomical sites associated with ILBC metastasis (ovaries, abdominal cavity, skin, bone) reads like a catalog of tissue compartments with a favorable steroid hormone supply. Estrogen concentrations are up to 1000-fold higher in ovarian tissue and peritoneal cavity fluid as compared with the body circulation 20. Moreover, estrogens are produced by mesenchymal cells of the dermis, adipose tissue and bone 21. Accordingly, ILBCs seem to metastasize to sites of estrogen production. The most convincing case supporting this notion has been documented by Arnould et al. 22. They have reported an ILBC metastasis within an estrogen-producing granulosa cell tumor of the ovary, which had developed under tamoxifen therapy 22. There is indirect evidence that the orbital fat pad produces steroid hormones to regulate tear film composition 23,24. Hence, the orbit may represent another, albeit less often occupied niche attracting disseminated ILBC cells with local estrogens. Alternatively, OMs from ILBC may simply extend to the orbit from nearby bone metastases or from occult meningeosis carcinomatosa.

In conclusion, ILBC is over-represented in patients suffering from OMs. The high frequency of ILBC in OMs illustrates the special metastatic behavior of this tumor entity, which is possibly related to local hormonal growth factors.

## Conflict of Interest

None declared.

## Supporting Information

**Figure S1.** Matched primary ILBCs of patients 3 and 4.

**Table S1.** Histological classification by different observers.

**Table S2.** Published Case reports on orbital metastasis.

**Table S3.** Reports on the clinical presentation of metastatic ILBC.
