# Supplementary figures and images for: High frequency of lobular breast cancer in distant metastases to the orbit
Source: Cancer Med. 2014 Oct 30;4(1):104–11. doi: 10.1002/cam4.331 (PMC4312124; doi:10.1002/cam4.331)

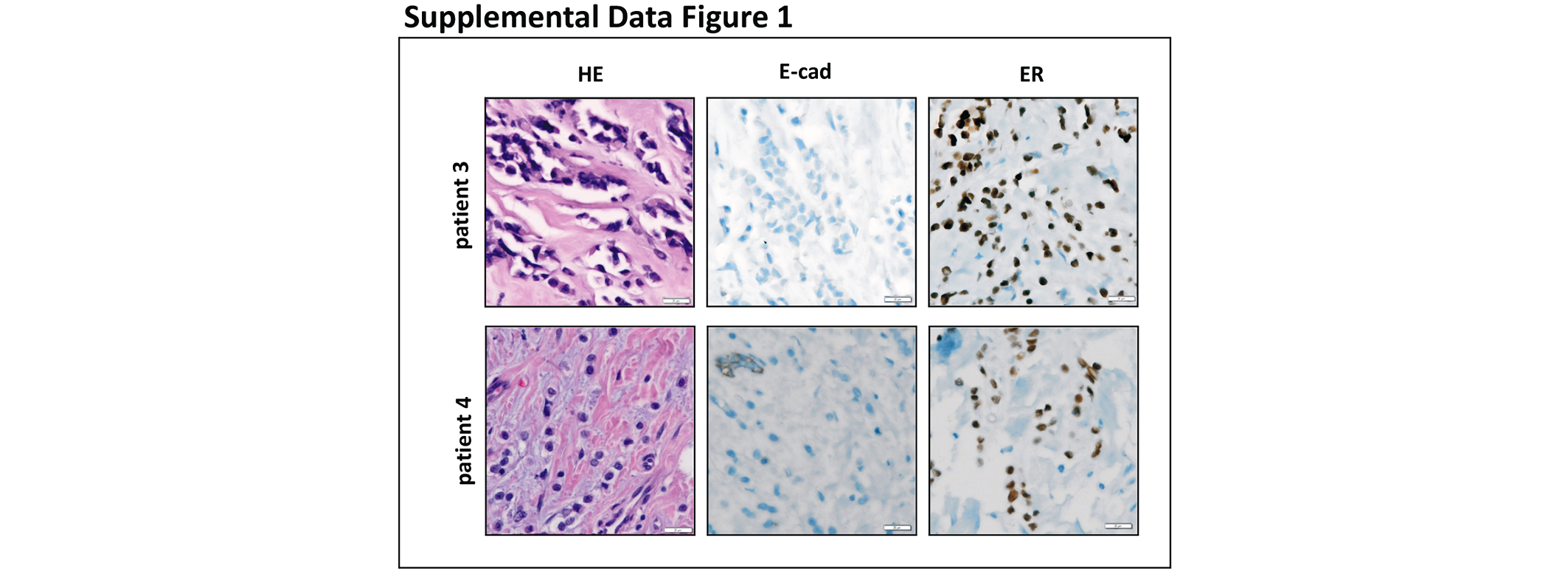

Supplement: Supplementary file 1 [file cam40004-0104-sd1.tif]
