# Supplementary material for: High frequency of lobular breast cancer in distant metastases to the orbit
Source: Cancer Med. 2014 Oct 30;4(1):104–11. doi: 10.1002/cam4.331 (PMC4312124; doi:10.1002/cam4.331)
Supplement: Supplementary file 2 [file cam40004-0104-sd2.doc]

|  | **Supplemental Data Table 1** | |  |  |  |  |
| --- | --- | --- | --- | --- | --- | --- |
|  |  | **histological characterization of breast cancers metastases to the orbit**  **by four different observers** | | | |  |
|  |  | **pathologist 1** | **pathologist 2** | **pathologist 3** | **pathologist 4** |  |
|  | **patient 1** | ILBC, G2 | ILBC, G2 | ILBC, G2 | ILBC, G2 |  |
|  | **patient 2** | ILBC, G2 | ILBC, G2 | ILBC, G2 | ILBC, G3 |  |
|  | **patient 3** | ILBC, G2 | ILBC, G3 | ILBC, G2 | ILBC, G2 |  |
|  | **patient 4** | ILBC, G3 | ILBC, G3 | pleo ILBC, G3 | ILBC, G3, solid |  |
|  | **patient 5** | ILBC, G3 | ILBC, G3 | pleo ILBC, G3 | ILBC, G3 |  |
|  | **patient 6** | ILBC, G2 | ILBC, G2 | ILBC, G2 | ILBC, G2 |  |
|  | **patient 7** | ILBC, G2 | ILBC, G2 | ILBC, G2 | ILBC, G2 |  |
|  | **patient 8** | IDBC, G3 | IDBC, G3 | pleo ILBC, G3 | IDBC, with SRC, G3 |  |
|  | IDBC, infiltrating ductal breast cancer; ILBC, infiltrating lobular breast cancer; pleo, pleomorhic;  SRC, signet ring cells | | | | |  |
