# Supplementary material for: High frequency of lobular breast cancer in distant metastases to the orbit
Source: Cancer Med. 2014 Oct 30;4(1):104–11. doi: 10.1002/cam4.331 (PMC4312124; doi:10.1002/cam4.331)
Supplement: Supplementary file 3 [file cam40004-0104-sd3.doc]

|  |  |  |  |  |  |  |
| --- | --- | --- | --- | --- | --- | --- |
|  | Supplemental Data Table 2 | | |  |  |  |
|  | **Case reports on orbital metastases (2008-2013)** | | | | |  |
|  | **patient** | **gender** | **age** | **ILBC (0 vs 1)** | **reference** |  |
|  | patient 1 | m | 55 | 0 | 1 |  |
|  | patient 2 | f | 53 | 0 | 2 |  |
|  | patient 3 | f | 73 | 0 | 3 |  |
|  | patient 4 | f | 73 | 0 | 4 |  |
|  | patient 5 | f | na | 0 | 5 |  |
|  | patient 6 | f | 70 | 0 | 6 |  |
|  | patient 7 | f | na | 0 | 7 |  |
|  | patient 8 | f | 66 | 0 | 8 |  |
|  | patient 9 | f | 83 | 0 | 9 |  |
|  | patient 10 | f | na | 0 | 10 |  |
|  | patient 11 | m | 24 | 0 | 11 |  |
|  | patient 12 | m | 77 | 0 | 12 |  |
|  | patient 13 | m | 63 | 0 | 13 |  |
|  | patient 14 | na | na | 0 | 14 |  |
|  | patient 15 | f | 58 | 0 | 15 | |
|  | patient 16 | f | 65 | 0 | 16 |  |
|  | patient 17 | m | na | 0 | 17 |  |
|  | patient 18 | m | 45 | 0 | 18 |  |
|  | patient 19 | m | 25 | 0 | 19 |  |
|  | patient 20 | m | 52 | 0 | 20 |  |
|  | patient 21 | m | 57 | 0 | 21 |  |
|  | patient 22 | f | 74 | 0 | 22 |  |
|  | patient 23 | m | na | 0 | 23 |  |
|  | patient 24 | f | 70 | 0 | 24 |  |
|  | patient 25 | m | 65 | 0 | 25 |  |
|  | patient 26 | f | 66 | 1 | 26 |  |
|  | patient 27 | f | 45 | 1 | 27 |  |
|  | patient 28 | f | 46 | 1 | 28 |  |
|  | patient 29 | f | 54 | 1 | 29 |  |
|  | patient 30 | f | 50 | 1 | 30 |  |
|  | patient 31 | f | 70 | 1 | 31 |  |
|  | patient 32 | f | 46 | 1 | 32 |  |
|  | patient 33 | f | 79 | 1 | 15 | |
|  | patient 34 | f | 55 | 1 | 33 |  |
|  | patient 35 | f | 50 | 1 | 34 |  |
|  | patient 36 | f | 98 | 1 | 35 |  |
|  | patient 37 | f | 51 | 0 | 36 |  |
|  | patient 38 | m | 80 | 0 | 37 |  |
|  | patient 39 | f | 70 | 0 | 38 |  |
|  | patient 40 | f | 72 | 0 | 39 |  |
|  | patient 41 | f | na | 0 | 40 |  |
|  | patient 42 | na | na | 0 | 41 |  |
|  | patient 43 | f | 72 | 0 | 42 |  |
|  | patient 44 | na | na | 0 | 43 |  |
|  | patient 45 | m | 62 | 0 | 44 |  |
|  | patient 46 | m | 49 | 0 | 1 |  |
|  | patient 47 | f | 53 | 0 | 45 |  |
|  | patient 48 | m | 16 | 0 | 46 |  |
|  | patient 49 | m | 81 | 0 | 47 |  |
|  | patient 50 | m | 56 | 0 | 48 |  |
|  | patient 51 | m | na | 0 | 49 |  |
|  | patient 52 | m | na | 0 | 50 |  |
|  | patient 53 | m | na | 0 | 51 |  |
|  | patient 54 | m | na | 0 | 52 |  |
|  | patient 55 | m | na | 0 | 52 |  |
|  | patient 56 | m | 54 | 0 | 53 |  |
|  | patient 57 | m | 72 | 0 | 54 |  |
|  | patient 58 | f | 66 | 0 | 55 |  |
|  | patient 59 | m | 74 | 0 | 56 |  |
|  | patient 60 | na | na | 0 | 57 |  |
|  | patient 61 | m | 48 | 0 | 58 |  |
|  | patient 62 | f | na | 0 | 59 |  |
|  | patient 63 | m | 61 | 0 | 60 |  |
|  | patient 64 | na | na | 0 | 61 |  |
|  | patient 65 | m | 75 | 0 | 62 |  |
|  | patient 66 | m | 46 | 0 | 63 |  |
|  | patient 67 | na | na | 0 | 64 |  |
|  | patient 68 | na | na | 0 | 64 |  |
|  | patient 69 | m | na | 0 | 65 |  |
|  | patient 70 | na | na | 0 | 66 |  |
|  | patient 71 | f | 53 | 0 | 67 |  |
|  | patient 72 | m | 66 | 0 | 68 |  |
|  |  |  |  |  |  |  |

## References for Supplemental Data Table 2

1. Ng E, Ilsen PF Orbital metastases. *Optometry*. 2010;81:647-57.

2. Dmuchowska DA, Krasnicki P, Obuchowska I, Kochanowicz J, Syta-Krzyzanowska A, Mariak Z Ophthalmic manifestation of skull base metastasis from breast cancer. *Med Sci Monit*. 2012;18:CS105-8.

3. Lucereau-Barbier M, El Falah S, Desoutter M, Job L, Ducasse A, et al. [Breast adenocarcinoma discovered by orbital metastases: a case report and review of the literature]. *J Gynecol Obstet Biol Reprod (Paris)*. 2012;41:96-9.

4. Eckardt AM, Rana M, Essig H, Gellrich NC Orbital metastases as first sign of metastatic spread in breast cancer: case report and review of the literature. *Head Neck Oncol*. 2011;3:37.

5. Janicijevic-Petrovic M, Sarenac T, Sreckovic S, Vulovic D, Janicijevic K Orbital metastases from breast cancer: a case report. *Bosn J Basic Med Sci*. 2011;11:253-5.

6. Papathanassiou M, Nikita E, Theodossiadis P, Vergados I Orbital metastasis secondary to breast cancer mimicking thyroid-associated ophthalmopathy. *Clin Exp Optom*. 2010;93:368-9.

7. Tanaka Y, Kurokawa T, Arita K, Kuramochi J, Usui S, et al. [A case of recurrent breast carcinoma metastasis successfully treated with S-1 and zoledronic acid therapy]. *Gan To Kagaku Ryoho*. 2009;36:97-9.

8. Kuo SC, Hsiao SC, Chiou CC, Chen FF, Huang KC Metastatic carcinoma of the breast: a case with the unusual presentation of unilateral periorbital edema. *Jpn J Ophthalmol*. 2008;52:305-7.

9. Milman T, Pliner L, Langer PD Breast carcinoma metastatic to the orbit: an unusually late presentation. *Ophthal Plast Reconstr Surg*. 2008;24:480-2.

10. Dhrami-Gavazi E, Lo C, Patel P, Galic V, Pareja F, Kazim M Gestational Choriocarcinoma Metastasis to the Extraocular Muscle: A Case Report. *Ophthal Plast Reconstr Surg*. 2013;In press.

11. Charles NC, Ng DD, Zoumalan CI Signet cell adenocarcinoma of the rectum metastatic to the orbit. *Ophthal Plast Reconstr Surg*. 2013;28:e1-2.

12. Chen SF, Yii CY, Chou JW Colon cancer with orbital metastasis. *Clin Gastroenterol Hepatol*. 2013;9:e76-7.

13. Okere PC, Tushar M Retro-orbital metastasis from differentiated thyroid carcinoma in a radioiodine therapy-naive patient: any lesson learned? *Med Princ Pract*. 2012;21:579-81.

14. Anoop TM, Mini PN, Divya KP, Nikhil S, Jabbar PK Clinical images. Thyroid follicular carcinoma presenting as intraorbital, intracranial, and subcutaneous metastasis. *Am J Surg*. 2010;199:e72-4.

15. Martorell-Calatayud A, Requena C, Diaz-Recuero JL, Haro R, Sarasa JL, et al. Mask-like metastasis: report of 2 cases of 4 eyelid metastases and review of the literature. *Am J Dermatopathol*. 2010;32:9-14.

16. Woo D, Leong J, Waring D, Sharma A, Martin P Orbital gastrointestinal stromal tumor metastasis. *Orbit*. 2012;31:129-31.

17. Piccirillo M, Granata V, Albino V, Palaia R, Setola SV, et al. Can hepatocellular carcinoma (HCC) produce unconventional metastases? Four cases of extrahepatic HCC. *Tumori*. 2013;99:e19-23.

18. Guerriero S, Infante G, Giancipoli E, Cocchi S, Fiore MG, et al. Hepatocellular Carcinoma Metastasis to the Orbit in a Coinfected HIV+ HBV+ Patient Previously Treated with Orthotopic Liver Transplantation: A Case Report. *Case Rep Ophthalmol Med*. 2011;2011:549270.

19. Mustapha SK, Madachi DA Orbital metastasis of hepatocellular carcinoma: a case report. *West Afr J Med*. 2011;30:305-7.

20. Quick AM, Bloomston M, Kim EY, Hall NC, Mayr NA Complete response to radiation therapy of orbital metastasis from hepatocellular carcinoma. *World J Gastroenterol*. 2009;15:6000-3.

21. Fonseca Junior NL, Frizon L, Paves L, Wolosker AM, Manso PG [An unusual orbital metastatic lesion: the only finding in a case of hepatocellular carcinoma: case report]. *Arq Bras Oftalmol*. 2008;71:865-7.

22. Hirunwiwatkul P, Tirakunwichcha S, Meesuaypong P, Shuangshoti S Orbital metastasis of hepatocellular carcinoma. *J Neuroophthalmol*. 2008;28:47-50.

23. Pitts J, Chang CH, Mavrikakis I, Shaikh A, Rootman J Hepatocellular carcinoma presenting as orbital bone metastasis. *Ophthal Plast Reconstr Surg*. 2008;24:477-9.

24. Fyrmpas G, Televantou D, Papageorgiou V, Nofal F, Constantinidis J Unsuspected breast carcinoma presenting as orbital complication of rhinosinusitis. *Eur Arch Otorhinolaryngol*. 2008;265:979-82.

25. Polito E, Pichierri P, Occhini R, Loffredo A, Moramarco A, Balestrazzi A Orbital metastasis associated with primary breast carcinoma in a man detected during peribulbar anesthesia for cataract surgery. *Eur J Ophthalmol*. 2008;18:1031-3.

26. Francone E, Murelli F, Paroldi A, Margarino C, Friedman D Orbital swelling as a first symptom in breast carcinoma diagnosis: a case report. *J Med Case Rep*. 2013;4:211.

27. Radovanovic AB, Rasic D, Buta M, Dzodic R Breast cancer metastasis to the conjunctiva. *Vojnosanit Pregl*. 2013;70:331-4.

28. Saffra N, Rakhamimov A, Wrzolek MA, Solomon WB, Cooper J, Borgen P Orbital Metastasis as the Initial Presentation in Bilateral Lobular Invasive Carcinoma of the Breast. *Ophthal Plast Reconstr Surg*. 2013;In press.

29. Volleamere A, Kirwan C, Bramley M Orbital metastases as the primary presentation of lobular breast cancer. *Breast J*. 2013;19:333-4.

30. Kim HJ, Wojno TH, Grossniklaus H Atypical bilateral orbital metastases of lobular breast carcinoma. *Ophthal Plast Reconstr Surg*. 2012;28:e142-3.

31. Tomizawa Y, Ocque R, Ohori NP Orbital metastasis as the initial presentation of invasive lobular carcinoma of breast. *Intern Med*. 2012;51:1635-8.

32. Gupta S, Bhatt VR, Varma S Unilateral orbital pain and eyelid swelling in a 46-year-old woman: orbital metastasis of occult invasive lobular carcinoma of breast masquerading orbital pseudotumour. *BMJ Case Rep*. 2011;bcr1220103580.

33. Kanoh T, Nakano Y, Inatome J, Sakamoto T, Kira T, et al. [A case of successfully treated orbital metastasis from breast cancer by radiation therapy]. *Gan To Kagaku Ryoho*. 2008;35:2231-3.

34. Kouvaris JR, Gkongkou PV, Papadimitriou CA, Papacharalampous XN, Antypas CE, et al. Bilateral metastases to extraocular muscles from lobular breast carcinoma. *Onkologie*. 2008;31:387-9.

35. Surace D, Piscioli I, Morelli L, Valduga F, Licci S Orbital metastasis as the first sign of "Dormant" breast cancer dissemination 25 years after mastectomy. *Jpn J Ophthalmol*. 2008;52:423-5.

36. Chen J, Wei R, Ma X Orbital metastasis of retroperitoneal leiomyosarcoma. *Med Oncol*. 2012;29:392-5.

37. Johnson D, Warder D, Plourde ME, Brundage M, ten Hove M Orbital metastasis secondary to merkel cell carcinoma: case report and literature review. *Orbit*. 2013;32:263-5.

38. Ben Hadj Hamida F, Fezani M, Ben Amor H, Krifa F, Chaabani L, et al. [Orbital metastasis from cutaneous melanoma]. *J Fr Ophtalmol*. 2009;32:425-9.

39. Ullah T, Gurwood AS, Myers MD Ocular metastasis of cutaneous malignant melanoma. *Optometry*. 2009;80:572-8.

40. Seiff BD, Seiff SR Orbital metastasis from medullary thyroid carcinoma. *Ophthal Plast Reconstr Surg*. 2008;24:484-5.

41. Sira M, Clauss RP, Maclean C, Rose GE Orbital metastases from neuroendocrine carcinoma, masquerading as graves orbitopathy. *Orbit*. 2010;29:94-6.

42. Matsuo T, Ichimura K, Tanaka T, Takenaka T, Nakayama T Neuroendocrine tumor (carcinoid) metastatic to orbital extraocular muscle: case report and literature review. *Strabismus*. 2010;18:123-8.

43. Lachhab L, Fikri M, Aitbenhaddou EH, Arkha Y, Regragui W, et al. [Atypical metastatic sites for adenocarcinoma of the lung]. *J Fr Ophtalmol*. 2013;36:e23-6.

44. Koma Y, Goto K, Yoshida C, Kimura K, Matsumoto Y, et al. Orbital metastasis secondary to pulmonary adenocarcinoma treated with gefitinib: a case report. *J Med Case Rep*. 2012;6:353.

45. Azad A Metastatic non-small cell lung cancer presenting with an orbital metastasis: a case report. *Cases J*. 2008;1:89.

46. Attili SV, Jain A, Saini KV, Batra U, Govind Babu K, et al. Orbital metastasis: a rare presentation of osteosarcoma. *Int Ophthalmol*. 2008;28:433-6.

47. Lefresne S, Fairchild A, Johnson R, Deschenes J, Russell L, Pederson J Genitourinary malignancy presenting as an ocular metastasis: A case report and review of the literature. *Can Urol Assoc J*. 6:E67-71.

48. Heintz J, Kahn B, Kramer A Prostate adenocarcinoma with orbital metastasis in an HIV+ man. *Can J Urol*. 2011;18:5660-2.

49. Rosado P, de Vicente JC, Vivanco B, de Villalain L, Santamarta T Clinical and immunohistochemical analysis of orbital metastasis from prostate carcinoma. *J Craniofac Surg*. 2011;22:2141-3.

50. Barahimi B, Patel A, Bilyk JR Orbital metastasis mimicking subperiosteal abscess. *Orbit*. 2010;29:165-7.

51. Nayyar R, Singh P, Panda S, Kashyap S, Gupta NP Proptosis due to "isolated" soft tissue orbital metastasis of prostate carcinoma. *Indian J Cancer*. 47:74-6.

52. Alsuhaibani AH, Carter KD, Nerad JA, Lee AG Prostate carcinoma metastasis to extraocular muscles. *Ophthal Plast Reconstr Surg*. 2008;24:233-5.

53. Pastor Navarro H, Donate Moreno MJ, Carrion Lopez P, Martinez Ruiz J, Lorenzo Romero JG, et al. [Retroorbital metastasis in the initial diagnosis of prostate cancer]. *Arch Esp Urol*. 2008;61:524-7.

54. Peng KL, Kao SC, Yang CF, Kau HC, Tsai CC, Hsu WM Metastatic prostatic adenocarcinoma to the orbit diagnosed by prostate-specific antigen staining. *Eye (Lond)*. 2008;22:320-2.

55. Rocha Filho FD, Lima GG, Ferreira FV, Lima MG, Hissa MN Orbital metastasis as primary clinical manifestation of thyroid carcinoma--case report and literature review. *Arq Bras Endocrinol Metabol*. 2008;52:1497-500.

56. Evgeniou E, Menon KR, Jones GL, Whittet H, Williams W Renal cell carcinoma metastasis to the paranasal sinuses and orbit. *BMJ Case Rep*. 2012;bcr0120125492.

57. Jung JW, Yoon SC, Han DH, Chi M Metastatic renal cell carcinoma to the orbit and the ethmoid sinus. *J Craniofac Surg*. 2012;23:e136-8.

58. Preechawai P, Amrith S, Yip CC, Goh KY Orbital metastasis of renal cell carcinoma masquerading as cysticercosis. *Orbit*. 2008;27:370-3.

59. Gosslee JM, Misra RP, Langford MP, Vekovius B, Byrd WA, Flynn SB Orbital metastasis of keratinizing squamous cell cervical carcinoma with giant cells. A case report. *Int Ophthalmol*. 2009;29:39-44.

60. Lee JY, Lee HJ, Jung MS, Kim SY Metastatic esophageal squamous cell carcinoma to the orbit and periorbit masquerading as periorbital abscess. *Korean J Ophthalmol*. 2010;24:123-5.

61. Tsai CK, Lui CC, Chang NJ, Hsu HC Anterior intraorbital metastasis from squamous cell carcinoma of the esophagus. *Jpn J Ophthalmol*. 2008;52:408-10.

62. Galm T, Kulkarni A, Ahmad I Laryngeal carcinoma metastasis to the orbit: case report. *J Laryngol Otol*. 2011;125:533-5.

63. Tezcan Y Bilateral orbital metastases from small cell lung cancer: a case report. *Acta Clin Belg*. 2013;68:59-61.

64. Henning M, Hu Q, Siegelmann-Danieli N Orbital metastasis as the presenting symptom of extensive stage small cell lung cancer. *Eur J Intern Med*. 2008;19:65-6.

65. Parikh JG, Burnstine MA, Kase S, Rao NA Salivary duct carcinoma metastatic to eyelid and orbit-a case report. *Graefes Arch Clin Exp Ophthalmol*. 2008;246:1185-8.

66. Ozpacaci T, Mulazimoglu M, Tamam MO, Leblebici C, Yildiz K, et al. Intraocular and orbital metastasis as a rare form of clinical presentation of insular thyroid cancer. *Ann Endocrinol (Paris)*. 2012;73:222-4.

67. SooHoo JR, Gonzalez MO, Siomos VJ, Durairaj VD Urothelial carcinoma with orbital metastasis. *Urology*. 2012;80:e45-6.

68. Wettach GR, Steele EA Urothelial cell carcinoma of the bladder presenting as orbital metastasis. *Arch Pathol Lab Med*. 2008;132:1224.
