# Supplementary material for: High frequency of lobular breast cancer in distant metastases to the orbit
Source: Cancer Med. 2014 Oct 30;4(1):104–11. doi: 10.1002/cam4.331 (PMC4312124; doi:10.1002/cam4.331)
Supplement: Supplementary file 4 [file cam40004-0104-sd4.doc]

|  |  |  |  |  |  |  |
| --- | --- | --- | --- | --- | --- | --- |
|  | **Supplemental Data Table 3** | | |  |  |  |
|  | **Reports on clinical presentation of metastatic ILBC (1998-2013)** | | | | |  |
|  | **patient** | **gender** | **age** | **metastasis localization** | **reference** |  |
|  | patient 1 | f | 72 | stomach | 1 |  |
|  | patient 2 | f | 61 | stomach | 1 |  |
|  | patient 3 | f | 58 | stomach | 1 |  |
|  | patient 4 | f | 55 | stomach | 1 |  |
|  | patient 5 | f | 47 | orbit | 2 |  |
|  | patient 6 | f | 53 | vulva | 3 |  |
|  | patient 7 | f | 68 | stomach | 4 |  |
|  | patient 8 | f | 53 | orbit | 5 |  |
|  | patient 9 | f | 45 | stomach | 6 |  |
|  | patient 10 | f | 77 | stomach | 7 |  |
|  | patient 11 | f | 82 | rectum | 8 |  |
|  | patient 12 | f | 63 | ovary | 9 |  |
|  | patient 13 | f | 61 | orbit | 10 |  |
|  | patient 14 | f | 50 | muscle | 11 |  |
|  | patient 15 | f | 78 | uterus | 12 |  |
|  | patient 16 | f | 76 | peritoneum | 13 |  |
|  | patient 17 | f | 62 | colon | 14 |  |
|  | patient 18 | f | 50 | cervix | 15 |  |
|  | patient 19 | f | 65 | aorta | 16 |  |
|  | patient 20 | f | 63 | uterus | 17 |  |
|  | patient 21 | f | 53 | orbit | 18 |  |
|  | patient 22 | f | 73 | stomach | 19 |  |
|  | patient 23 | f | 67 | rectum | 20 |  |
|  | patient 24 | f | 51 | esophagus | 21 |  |
|  | patient 25 | f | 66 | esophagus | 21 |  |
|  | patient 26 | f | 80 | stomach | 21 |  |
|  | patient 27 | f | na | orbit ,eye lid | 22 |  |
|  | patient 28 | f | 46 | peritoneum | 23 |  |
|  | patient 29 | f | 75 | ileum | 24 |  |
|  | patient 30 | f | 69 | cervix | 25 |  |
|  | patient 31 | f | 54 | stomach | 26 |  |
|  | patient 32 | f | 61 | stomach | 26 |  |
|  | patient 33 | f | 57 | muscle | 27 |  |
|  | patient 34 | f | 65 | liver | 28 |  |
|  | patient 35 | f | 70 | stomach | 29 |  |
|  | patient 36 | f | 55 | orbit | 30 |  |
|  | patient 37 | f | 98 | orbit | 31 |  |
|  | patient 38 | f | 55 | orbit | 32 |  |
|  | patient 39 | f | 53 | stomach | 33 |  |
|  | patient 40 | f | 56 | ovary | 34 |  |
|  | patient 41 | f | 78 | uterus | 35 |  |
|  | patient 42 | f | 61 | stomach | 36 |  |
|  | patient 43 | f | 92 | anus | 37 |  |
|  | patient 44 | f | 58 | duodenum | 38 |  |
|  | patient 45 | f | ns | pleura | 39 |  |
|  | patient 46 | f | 64 | skin | 40 |  |
|  | patient 47 | f | 82 | rectum | 41 | |
|  | patient 48 | f | 64 | meninges | 42 |  |
|  | patient 49 | f | 79 | orbit, eyelids | 43 | |
|  | patient 50 | f | 64 | ovary | 44 |  |
|  | patient 51 | f | 47 | peritoneum | 45 |  |
|  | patient 52 | f | 53 | rectal | 45 |  |
|  | patient 53 | f | 70 | stomach, rectum | 46 |  |
|  | patient 54 | f | 79 | colon | 47 |  |
|  | patient 55 | f | 68 | parotid gland | 48 |  |
|  | patient 56 | f | 46 | orbit | 49 |  |
|  | patient 57 | f | 70 | orbit | 50 |  |
|  | patient 58 | f | 50 | orbit | 51 |  |
|  | patient 59 | f | 45 | orbit | 52 |  |
|  | patient 60 | f | 46 | orbit | 53 |  |
|  | patient 61 | f | 58 | kidney | 54 |  |
|  | patient 62 | f | 54 | orbit | 55 |  |
|  | patient 63 | f | 66 | orbit | 56 |  |
|  |  |  |  |  |  |  |

## References for Supplemental Data Table 3

1. Schwarz RE, Klimstra DS, Turnbull AD Metastatic breast cancer masquerading as gastrointestinal primary. *Am J Gastroenterol*. 1998;93:111-4.

2. Toller KK, Gigantelli JW, Spalding MJ Bilateral orbital metastases from breast carcinoma. A case of false pseudotumor. *Ophthalmology*. 1998;105:1897-901.

3. Menzin AW, De Risi D, Smilari TF, Kalish PE, Vinciguerra V Lobular breast carcinoma metastatic to the vulva: a case report and literature review. *Gynecol Oncol*. 1998;69:84-8.

4. Van Trappen P, Serreyn R, Elewaut AE, Cocquyt V, Van Belle S Abdominal pain with anorexia in patients with breast carcinoma. *Ann Oncol*. 1998;9:1243-5.

5. Wolstencroft SJ, Hodder SC, Askill CF, Sugar AW, Jones EW, Griffiths AP Orbital metastasis due to interval lobular carcinoma of the breast: a potential mimic of lymphoma. *Arch Ophthalmol*. 1999;117:1419-21.

6. Pera M, Riera E, Lopez R, Vinolas N, Romagosa C, Miquel R Metastatic carcinoma of the breast resembling early gastric carcinoma. *Mayo Clin Proc*. 2001;76:205-7.

7. Reiman T, Butts CA Upper gastrointestinal bleeding as a metastatic manifestation of breast cancer: a case report and review of the literature. *Can J Gastroenterol*. 2001;15:67-71.

8. Bamias A, Baltayiannis G, Kamina S, Fatouros M, Lymperopoulos E, et al. Rectal metastases from lobular carcinoma of the breast: report of a case and literature review. *Ann Oncol*. 2001;12:715-8.

9. Arnould L, Franco N, Soubeyrand MS, Mege F, Belichard C, et al. Breast carcinoma metastasis within granulosa cell tumor of the ovary: morphologic, immunohistologic, and molecular analyses of the two different tumor cell populations. *Hum Pathol*. 2002;33:445-8.

10. Reeves D, Levine MR, Lash R Nonpalpable breast carcinoma presenting as orbital infiltration: case presentation and literature review. *Ophthal Plast Reconstr Surg*. 2002;18:84-8.

11. El Khoury M, Cherel P, Becette V, De Maulmont C, Costes V, et al. Unusual soft-tissue metastasis of an invasive lobular carcinoma mimicking fasciitis. *AJR Am J Roentgenol*. 2004;182:745-7.

12. Famoriyo A, Sawant S, Banfield PJ Abnormal uterine bleeding as a presentation of metastatic breast disease in a patient with advanced breast cancer on tamoxifen therapy. *Arch Gynecol Obstet*. 2004;270:192-3.

13. Mylonas I, Janni W, Friese K, Gerber B Unexpected metastatic lobular carcinoma of the breast with intraabdominal spread and subsequent port-site metastasis after diagnostic laparoscopy for exclusion of ovarian cancer. *Gynecol Oncol*. 2004;95:405-8.

14. Signorelli C, Pomponi-Formiconi D, Nelli F, Pollera CF Single colon metastasis from breast cancer: a clinical case report. *Tumori*. 2005;91:424-7.

15. Haji BE, Kapila K, Francis IM, Temmim L, Ahmed MS Cytomorphological features of metastatic mammary lobular carcinoma in cervicovaginal smears: report of a case and review of literature. *Cytopathology*. 2005;16:42-8.

16. Giagounidis AA, Heinsch M, von Barany RU, Erlemannb R, Aul C Breast cancer metastasis to the aortic vessel wall. *Onkologie*. 2005;28:369.

17. Erkanli S, Kayaselcuk F, Kuscu E, Bolat F, Sakalli H, Haberal A Lobular carcinoma of the breast metastatic to the uterus in a patient under adjuvant anastrozole therapy. *Breast*. 2006;15:558-61.

18. Kadivar M, Joulaee A, Kashkouli MB, Kharazi HH, Kalantari M, Kumar PV Orbital metastasis as the first presentation of nonpalpable invasive lobular carcinoma of the breast. *Breast J*. 2006;12:75-6.

19. Aurello P, D'Angelo F, Cosenza G, Petrocca S, Stoppacciaro A, et al. Gastric metastasis 14 years after mastectomy for breast lobular carcinoma: case report and literature review. *Am Surg*. 2006;72:456-60.

20. Franceschini G, Manno A, Mule A, Verbo A, Rizzo G, et al. Gastro-intestinal symptoms as clinical manifestation of peritoneal and retroperitoneal spread of an invasive lobular breast cancer: report of a case and review of the literature. *BMC Cancer*. 2006;6:193.

21. Nazareno J, Taves D, Preiksaitis HG Metastatic breast cancer to the gastrointestinal tract: a case series and review of the literature. *World J Gastroenterol*. 2006;12:6219-24.

22. Kuchel JM, Bowling JC Bilateral lower eyelid masses. Orbital (intraorbital and extraocular) metastases in breast cancer. *Arch Dermatol*. 2006;142:1351-6.

23. Kobayashi T, Adachi S, Matsuda Y, Tominaga S A case of metastatic lobular breast carcinoma with detection of the primary tumor after ten years. *Breast Cancer*. 2007;14:333-6.

24. Al-Qahtani MS Gut metastasis from breast carcinoma. *Saudi Med J*. 2007;28:1590-2.

25. Perisic D, Jancic S, Kalinovic D, Cekerevac M Metastasis of lobular breast carcinoma to the cervix. *J Obstet Gynaecol Res*. 2007;33:578-80.

26. Jones GE, Strauss DC, Forshaw MJ, Deere H, Mahedeva U, Mason RC Breast cancer metastasis to the stomach may mimic primary gastric cancer: report of two cases and review of literature. *World J Surg Oncol*. 2007;5:75.

27. Balibrea JM, Cantero R, Garcia-Calvo M, Garcia-Perez JC, Furio-Bacete V, et al. Perianal metastases from lobular breast carcinoma. *Clin Transl Oncol*. 2007;9:606-9.

28. Wu JM, Fackler MJ, Halushka MK, Molavi DW, Taylor ME, et al. Heterogeneity of breast cancer metastases: comparison of therapeutic target expression and promoter methylation between primary tumors and their multifocal metastases. *Clin Cancer Res*. 2008;14:1938-46.

29. Ciulla A, Castronovo G, Tomasello G, Maiorana AM, Russo L, et al. Gastric metastases originating from occult breast lobular carcinoma: diagnostic and therapeutic problems. *World J Surg Oncol*. 2008;6:78.

30. Kouvaris JR, Gkongkou PV, Papadimitriou CA, Papacharalampous XN, Antypas CE, et al. Bilateral metastases to extraocular muscles from lobular breast carcinoma. *Onkologie*. 2008;31:387-9.

31. Surace D, Piscioli I, Morelli L, Valduga F, Licci S Orbital metastasis as the first sign of "Dormant" breast cancer dissemination 25 years after mastectomy. *Jpn J Ophthalmol*. 2008;52:423-5.

32. Kanoh T, Nakano Y, Inatome J, Sakamoto T, Kira T, et al. [A case of successfully treated orbital metastasis from breast cancer by radiation therapy]. *Gan To Kagaku Ryoho*. 2008;35:2231-3.

33. Neal L, Sookhan N, Reynolds C Occult breast carcinoma presenting as gastrointestinal metastases. *Case Report Med*. 2009;2009:564756.

34. Ustaalioglu BB, Bilici A, Seker M, Salman T, Gumus M, et al. Metastasis of lobular breast carcinoma to the uterus in a patient under anastrozole therapy. *Onkologie*. 2009;32:424-6.

35. Bogliolo S, Morotti M, Valenzano Menada M, Fulcheri E, Musizzano Y, Casabona F Breast cancer with synchronous massive metastasis in the uterine cervix: a case report and review of the literature. *Arch Gynecol Obstet*. 2010;281:769-73.

36. Vennapusa B, Oman SA, Parasher G, Cerilli LA C-kit-positive gastric metastasis of lobular carcinoma of the breast masquerading as gastrointestinal stromal tumor. *Breast Cancer*. 2009;17:303-5.

37. Puglisi M, Varaldo E, Assalino M, Ansaldo G, Torre G, Borgonovo G Anal metastasis from recurrent breast lobular carcinoma: a case report. *World J Gastroenterol*. 2009;15:1388-90.

38. Nihon-Yanagi Y, Park Y, Ooshiro M, Aoki H, Suzuki Y, et al. A case of recurrent invasive lobular carcinoma of the breast found as metastasis to the duodenum. *Breast Cancer*. 2009;16:83-7.

39. Shah SP, Morin RD, Khattra J, Prentice L, Pugh T, et al. Mutational evolution in a lobular breast tumour profiled at single nucleotide resolution. *Nature*. 2009;461:809-13.

40. Christgen M, Bruchhardt H, Hadamitzky C, Rudolph C, Steinemann D, et al. Comprehensive genetic and functional characterization of IPH-926: a novel CDH1-null tumour cell line from human lobular breast cancer. *J Pathol*. 2009;217:620-32.

41. Lopez Deogracias M, Flores Jaime L, Arias-Camison I, Zamacola I, Murillo Guibert J, et al. Rectal metastasis from lobular breast carcinoma 15 years after primary diagnosis. *Clin Transl Oncol*. 2010;12:150-3.

42. Grewal J, Zhou H, Factor R, Kesari S Isolated loss of hormonal receptors in leptomeningeal metastasis from estrogen receptor- and progesterone receptor-positive lobular breast cancer. *J Clin Oncol*. 2010;28:e200-2.

43. Martorell-Calatayud A, Requena C, Diaz-Recuero JL, Haro R, Sarasa JL, et al. Mask-like metastasis: report of 2 cases of 4 eyelid metastases and review of the literature. *Am J Dermatopathol*. 2010;32:9-14.

44. Engelstaedter V, Mylonas I Lower genital tract metastases at time of first diagnosis of mammary invasive lobular carcinoma. *Arch Gynecol Obstet*. 2011;283 Suppl 1:93-5.

45. Saranovic D, Kovac JD, Knezevic S, Susnjar S, Stefanovic AD, et al. Invasive lobular breast cancer presenting an unusual metastatic pattern in the form of peritoneal and rectal metastases: a case report. *J Breast Cancer*. 2011;14:247-50.

46. Arrangoiz R, Papavasiliou P, Dushkin H, Farma JM Case report and literature review: Metastatic lobular carcinoma of the breast an unusual presentation. *Int J Surg Case Rep*. 2011;2:301-5.

47. Mistrangelo M, Cassoni P, Castellano I, Codognotto E, Sapino A, et al. Obstructive colon metastases from lobular breast cancer: report of a case and review of the literature. *Tumori*. 2011;97:800-4.

48. Ando K, Masumoto N, Sakamoto M, Teraoka K, Suzuki T, et al. Parotid Gland Metastasis of Breast Cancer: Case Report and Review of the Literature. *Breast Care (Basel)*. 2011;6:471-73.

49. Gupta S, Bhatt VR, Varma S Unilateral orbital pain and eyelid swelling in a 46-year-old woman: orbital metastasis of occult invasive lobular carcinoma of breast masquerading orbital pseudotumour. *BMJ Case Rep*. 2011;bcr1220103580.

50. Tomizawa Y, Ocque R, Ohori NP Orbital metastasis as the initial presentation of invasive lobular carcinoma of breast. *Intern Med*. 2012;51:1635-8.

51. Kim HJ, Wojno TH, Grossniklaus H Atypical bilateral orbital metastases of lobular breast carcinoma. *Ophthal Plast Reconstr Surg*. 2012;28:e142-3.

52. Radovanovic AB, Rasic D, Buta M, Dzodic R Breast cancer metastasis to the conjunctiva. *Vojnosanit Pregl*. 2013;70:331-4.

53. Saffra N, Rakhamimov A, Wrzolek MA, Solomon WB, Cooper J, Borgen P Orbital Metastasis as the Initial Presentation in Bilateral Lobular Invasive Carcinoma of the Breast. *Ophthal Plast Reconstr Surg*. 2013;In press.

54. Al-Jarrah A, Taranikanti V, Sawhney S, Furrukh M, Al-Hosni M, et al. Metastatic invasive lobular carcinoma of the breast masquerading as a primary renal malignancy. *Sultan Qaboos Univ Med J*. 2013;13:460-2.

55. Volleamere A, Kirwan C, Bramley M Orbital metastases as the primary presentation of lobular breast cancer. *Breast J*. 2013;19:333-4.

56. Francone E, Murelli F, Paroldi A, Margarino C, Friedman D Orbital swelling as a first symptom in breast carcinoma diagnosis: a case report. *J Med Case Rep*. 2013;4:211.
